# The proteins cleaved by endogenous tryptic proteases in normal EDTA plasma by C18 collection of peptides for liquid chromatography micro electrospray ionization and tandem mass spectrometry

**DOI:** 10.1186/s12014-017-9174-9

**Published:** 2017-12-02

**Authors:** Jaimie Dufresne, Angelique Florentinus-Mefailoski, Juliet Ajambo, Ammara Ferwa, Peter Bowden, John Marshall

**Affiliations:** 1 Ryerson Analytical Biochemistry Laboratory, Ryerson University, 350 Victoria Street, Toronto, ON M5B 2K3 Canada; 2 Integrated BioBank of Luxembourg (IBBL), Luxembourg Institute of Health (LIH), 6 Rue Nicolas Ernest Barblé, 1210 Luxembourg, Luxembourg

## Abstract

The tryptic peptides from ice cold versus room temperature plasma were identified by C18 liquid chromatography and micro electrospray ionization tandem mass spectrometry (LC–ESI–MS/MS). Samples collected on ice showed low levels of endogenous tryptic peptides compared to the same samples incubated at room temperature. Plasma on ice contained peptides from albumin, complement, and apolipoproteins and others that were observed by the X!TANDEM and SEQUEST algorithms. In contrast to ice cold samples, after incubation at room temperature, greater numbers of tryptic peptides from well characterized plasma proteins, and from cellular proteins were observed. A total of 583,927 precursor ions and MS/MS spectra were correlated to 94,669 best fit peptides that reduced to 22,287 correlations to the best accession within a gene symbol and to 7174 correlations to at least 510 gene symbols with ≥ 5 independent MS/MS correlations (peptide counts) that showed FDR q-values ranging from E−9 (i.e. FDR = 0.000000001) to E−227. A set of 528 gene symbols identified by X!TANDEM and SEQUEST including C4B showed ≥ fivefold variation between ice cold versus room temperature incubation. STRING analysis of the protein gene symbols observed from endogenous peptides in normal plasma revealed an extensive protein-interaction network of cellular factors associated with cell signalling and regulation, the formation of membrane bound organelles, cellular exosomes and exocytosis network proteins. Taken together the results indicated that a pool of cellular proteins, or protein complexes, in plasma are apparently not stable and degrade soon after incubation at room temperature.

**Electronic supplementary material:**

The online version of this article (10.1186/s12014-017-9174-9) contains supplementary material, which is available to authorized users.

## Background

Many of the well-known proteins of human blood [1, 2] are cleaved by endogenous tryptic endopeptidases to release fully tryptic peptides that may be identified by collection over C18 followed by liquid chromatography, electrospray ionization and tandem mass spectrometry (LC–ESI–MS/MS) with a Paul ion trap or Qq-TOF [3–5]. Exopeptidases are active in blood fluid that give rise to non-tryptic peptides that are computationally challenging to identify [6–8]. However, incubating blood fluid samples at room temperature also releases tryptic peptide**s** that have been confirmed by high mass accuracy measurements of the parent peptide masses and the use of tryptic protease inhibitors [5, 8]. The endogenous peptides of normal plasma may contain fragments of commonly known blood proteins released from tissues, cells and organs that might be cleaved by proteases ex vivo [3–5]. Thus, the representation of proteins by endogenous peptides may not be closely related to protein concentration but rather to the stability of the protein to ex vivo proteolytic attack. Human plasma contains many highly abundant proteins such as albumin, apolipoproteins, protease inhibitors and others that digest efficiently with the exogenous addition of trypsin which masks the detection of low abundance proteins by LC–ESI–MS/MS [3, 9]. The secretion or release of cellular proteins into extracellular space may result in the preferential cleavage of the cellular proteins upon exposure to extracellular protease activity that provides simple experimental access to the cellular factors of plasma. Incubating whole blood at room temperature is known to result in cell mediated degradation [10]. Sampling artefacts prior to freezing may be a major source of pre-analytical variation [8, 11–27]. To date there has been considerable variation in the peptides observed, and even the trends reported, in the degradation of blood proteins between groups, likely from the large variation in sample collection and processing times [8, 11–27]. Here sampling variation was addressed by collecting EDTA plasma directly onto ice and then purposefully degrading samples at room temperature under controlled conditions.

A comparison to random MS/MS spectra based on a computerized Random spectra generator (RSG) or instrument noise was used to estimate the type I error rate and showed that the peptides identified per protein by the X!TANDEM algorithm were well separated from the random expectation curve [28, 29]. The probability that the peptide-to-protein distribution of the authentic plasma proteins identified by X!TANDEM were the same as that of random expectation was estimated to be less than one chance in 10,000 (p < 0.0001) (< E−4) [28–32]. The peptide-to-protein counts and peptide intensity values may be compared by analysis with SQL and a generic statistical software system such as S, SAS or R between treatments and controls [30–34]. Comparing the Gene Ontology category distributions of the observed blood proteins to that of the entire protein library is an independent way to examine the likelihood that the proteins observed were simply a random assemblage of molecules [35, 36]. Since noise or near random mis-correlations may result in the identification of giant proteins such as Titin (TTN), Nebulin (NEB) and others at a low frequency in all experimental treatments, an additional way to filter out background noise or random correlations is to compare proteins in one treatment group against control treatments so that errors from noise or contamination that should be shared by all groups can be eliminated [37, 38]. Here, the endogenous tryptic peptides of normal human EDTA plasma collected on ice or incubated at room temperature were extracted by C18 and analyzed by liquid chromatography with micro electrospray ionization and a linear quadrupole ion trap, correlated by X!TANDEM and SEQUEST and analyzed by SQL, R and STRING.

## Methods

The 1100 HPLC system was from Agilent (Santa Clara, CA, USA). The LTQ XL linear ion traps were obtained from Thermo Electron Corporation (Waltham, MA, USA). The random spectra generator was slightly modified from that provided by Zhu et al. [28]. The EDTA blood sample tubes were from Becton Dickinson (B367844 K2EDTA, 7.2 mg) (Franklin Lakes, NJ, USA). The C18 with 5 micron particle size and 300 Angstrom pore size was from Agilent Zorbax 300 SB-C18 5-micron (Agilent). The HPLC grade water and acetonitrile was from Caledon laboratories and the formic acid from Fluka (Georgetown, ON, Canada). Sequencing grade trypsin was from Roche (Basel, Switzerland). The ZipTip C18 micro preparative column (Millipore C18 ZipTips, cat # ZTC 18S 096, peptide capacity 5 µg) were from Millipore (Billerica, MA, USA).

### Plasma sample collection

Human plasma from 30 normal controls both male and female were collected and aliquoted by JGM under a Comité National d’Ethique de Recherche (CNER) Protocol #201107 “Biospecimen Research” at the Centre Hospitalier de Luxembourg. The plasma was collected in EDTA tubes (B367844 K2EDTA, 7.2 mg, Becton Dickenson) that were rapidly inverted 10 times before packing in ice. The ice-cold plasma was then separated from blood cells at 12,000 RCF for 20 min in a centrifuge set at 4 °C prior to aliquoting the plasma to 225 µL and storing in a − 80 °C freezer. The plasma from one or a few patients per week was sampled by JGM over the course of several months and each plasma sample typically required about ~ 1 h of preparation time from blood collection on ice, and ice-cold centrifugation to obtain plasma, aliquoting and freezing or freeze drying on each of the many sampling days.

### Experimental treatments

In an effort to sample the peptides that might degrade in normal human EDTA plasma in circulation, 82 random samples were collected and aliquoted on ice (ICE) and never warmed to room temperature before sampling to prevent the action of proteases ex vivo. In contrast, a set of 88 normal plasma samples were subsequently incubated at room temperature (RT) and sampled from 0 h over 72 h at room temperature to produce the complete range of peptides that might be cleaved in normal plasma under clinical conditions. In total, 170 peptide samples were prepared separately over C18 and then individually analyzed by LC–ESI–MS/MS. The results of the incubation and sampling from 0 to 72 h at room temperature, alongside preserved samples frozen in liquid nitrogen, frozen at − 80 °C and control samples incubated on ice from 0 to 72 h were combined for statistical analysis.

### Manual C18 solid phase extraction and injection

A total of 25 μL of the plasma sample was diluted in 200 μL of 5% formic acid for peptide extraction over a 0.6 µL C18 preparative column (Millipore C18 ZipTips, cat # ZTC 18S 096, peptide capacity 5 ug) [7, 8]. The resin was wet 10× with 5% formic acid and 65% acetonitrile, the resin was equilibrated 5× with 5% formic acid, the sample was collected, concentrated and desalted over the resin and eluted in 2 µL (> 3 resin volumes) of 5% formic acid and 65% acetonitrile according to the manufacturer’s instructions. The 2 µL eluent was immediately diluted with 18 µL of 5% formic acid for injection via a 20 µL loop with a Rheodyne manual injector.

### LC–ESI–MS/MS

The mass spectrometer was cleaned and calibrated with the manufacturer’s standard mixture, tuned to the Glu Fibrinogen and Angiotensin peptides, and tested for sensitivity by the infusion of GluFib and Angiotensin prior to each block of samples. Three linear ion trap LC–ESI–MS/MS systems were fitted with naive 300-micron ID silica C18 columns (Zorbax 300 SB-C18 5-micron, cat.no.899999-777, Agilent). The columns were conditioned and quality control tested with a digested mixture of alcohol dehydrogenase, cytochrome c and glycogen phosphorylase to confirm the system was working normally versus historical benchmarks [30]. The sensitivity of the system was tested with a BSA digest on the conditioned columns that showed a sensitivity for automatic identification by SEQUEST to ~ 1 fmol on column. The sample was introduced via a 20 µL sample loop and blank samples were injected into a 2 µL/min flow of 5% acetonitrile and 0.1% acetic acid. The gradient of acetonitrile was commenced after 12 min from 15 to 45% ACN over the course of 60 min and then to 65% ACN over 30 min, cleaning at 65% for 5 min before returning to 5% ACN. A roughly equal number of ice versus room temperature samples were analyzed on each of the C18 analytical columns in random order independently a few weeks apart. The column was washed extensively with 50% ACN until a clean background was obtained between samples. A total of ~ 5 µg of extracted and purified peptides from a 0.6 µL ZipTip was manually injected for each analytical HPLC separation at 2 µL/min over a 300 micron ID column (15 cm) with inline filter frits. The peptides were ionized at 4.5 kV via a micro electrospray ion source with 10 L/min of dry N_2_ gas with a transfer capillary temperature of 200 °C. The precursor ions were randomly and independently sampled (with up to 4 MS/MS spectra samples per precursor) without replacement as the peptides eluted from the HPLC from 350 to 2000 m/z with a Thermo Electron Corporation LTQ XL ion trap mass spectrometer [39]. There was no dynamic exclusion employed.

### Peptide MS/MS spectra correlation analysis

A federated library of 154,208 human proteins that differed by at least one amino acid was assembled from NCBI, Ensembl and Swiss Prot and made non-redundant using Structured Query Language (SQL). About 74% of the accession numbers in the human FASTA library have defined gene symbols. A physical filter of at least one thousand (E3) intensity counts for precursor ions was used to limit type I error [28]. The MS and MS/MS spectra of peptides recorded were correlated to the federated library with fully tryptic enzyme specification, a charge state of 2^+^ or 3^+^ with up to three missed cleavages with ± 3 m/z and the fragments within 0.5 Da [28–31, 33] using the X!TANDEM [40] and SEQUEST [41] algorithms.

### Computational analysis in SQL and statistical analysis with R

The random and independently sampled parent and fragment m/z and intensity values from MS and MS/MS spectra and the resulting peptide and protein identifications were parsed into an SQL database [33]. The SQL database utilized a complex key also known as an SHA1-HASH for each MS/MS peptide-protein identification to ensure that only the best fit of each MS/MS spectra at only one charge state was accepted, and thus no MS/MS spectra was assigned to more than one peptide sequence. The peptide-to-protein counts of serum samples were previously statistically analyzed using the generic statistical system S [8] or SAS [28, 30, 31, 33, 34] but in this study the data was analyzed using the generic open-source R statistical system [37]. The total peptide-to-protein fits (MS/MS correlation to peptides in proteins) of the authentic experimental data were compared to that of a computer generated set of random MS/MS spectra to compute the probability of type I error over the entire experiment using the goodness of fit test [28–34]. The distributions and quantile plots of the peptide-to-protein counts, log10 intensity, expectation values (E), peptide mass and delta mass values calculated by X!TANDEM were computed in R [30–32]. The peptide p-values from X!TANDEM were used to compute the cumulative p-values for peptide sequences and gene symbols [33]. The corresponding FDR q-values were computed by the method of Benjamini and Hochberg [42].

### STRING analysis

Protein identifications made by LC–ESI–MS/MS of human blood may be tested by comparing the distribution of protein descriptive terms over categories using goodness of fit analysis [36] or looking for protein networks and interactions with respect to random expectation [43]. The STRING algorithm V10 (Search Tool for the Retrieval of Interacting Genes/Proteins) was used to create a network of plasma proteins from the set of proteins with at least 5 tryptic peptides [35]. The probability distribution of proteins over Gene Ontology (GO) terms, as well as their protein interactions from the Kyoto Encyclopedia of Genes and Genomes (KEGG) protein interactions were calculated with respect to random expectation using the corrections provided by Bonferroni, or Benjamini and Hochberg [42] for the number of hypotheses tested.

## Results

### Plasma versus random, noise and dust controls

The best fit peptides from X!TANDEM and SEQUEST were collected together in SQL Server. Plasma collected on ice showed tryptic peptides from many well-known proteins C4A, C4B, C3, KNG1, ITIH4, ALB, FGA, APOA4 from both the X!TANDEM and SEQUEST algorithms. The X!TANDEM and SEQUEST algorithms further identified many proteins, and protein complexes, of an apparent cellular origin. Comparison to random MS/MS spectra, source noise, and dust were used to control type I error [7, 28, 29, 44]. The SEQUEST results were apparently contaminated by giant proteins [33] such as TTN (34,350 aa), NEB (8560 aa), CCDC168 (7081 aa), SYNE1 (8797 aa), OBSCN (6620 aa), TROPH (6907), SYNE2 (6885), MACF1 (7388 aa), AHNAK (5890) and others such as DST, LRP1, XIRP2, MUC16, FSIP2, LRP1B, PLEC that were identified in random spectra controls and are likely random mis-correlations [28, 29]. In addition to TTN, the analysis of source noise from blank runs indicated that ZNF503, VWCE, API5, TMEM199, SYT7, TPSG1, USP9X, DACT3, SUGP2, CXorf31, MYO3A, KLRC4, CYCS, RGP1, SYN1, DSPP, IMMP1L CAMK1, ASPM, AHNAK, among others were correlated by SEQUEST from noise spectra collected from LC–ESI–MS/MS gradient runs with blank samples [28, 29]. Furthermore, a tryptic digest of laboratory dust may contain keratins, POTEI, POTEE, POTEF, ACTA2, ACTB, ACTG2, GFAP, POTEM, MUC5AC and others in addition to those found in solvent gradients alone [7]. In contrast to SEQUEST, relatively few peptides from TTN were observed by X!TANDEM. Furthermore, X!TANDEM identified many cellular proteins from endogenous peptides in EDTA plasma from the ion trap that showed good agreement with the proteins from exogenous tryptic digestion of blood fluids and in agreement with previous results of endogenous peptides from the Paul 3D ion trap [3]. After discounting apparently spurious proteins from the negative controls of random, noise (solvents alone) or typically digested dust controls, the many cellular proteins identified by X!TANDEM and SEQUEST show excellent agreement with the many cellular proteins identified from serum or plasma by independent chromatographic separation and exogenous tryptic digestion [3, 33]. In agreement with previous results from exogenous digest by trypsin [44], endogenous peptides were observed from proteins in the ~ 1 ng/ml range such as creatine kinase CKB that exists in 1–5 ng/mL, acid phosphatase ACP in the 1–5 ng/ml range and carcinoembryonic antigen CEACAM5 in the 0.5–5 ng/ml concentration range.

### Statistical analysis of X!TANDEM results at the level of peptides

**Fig. 1:**
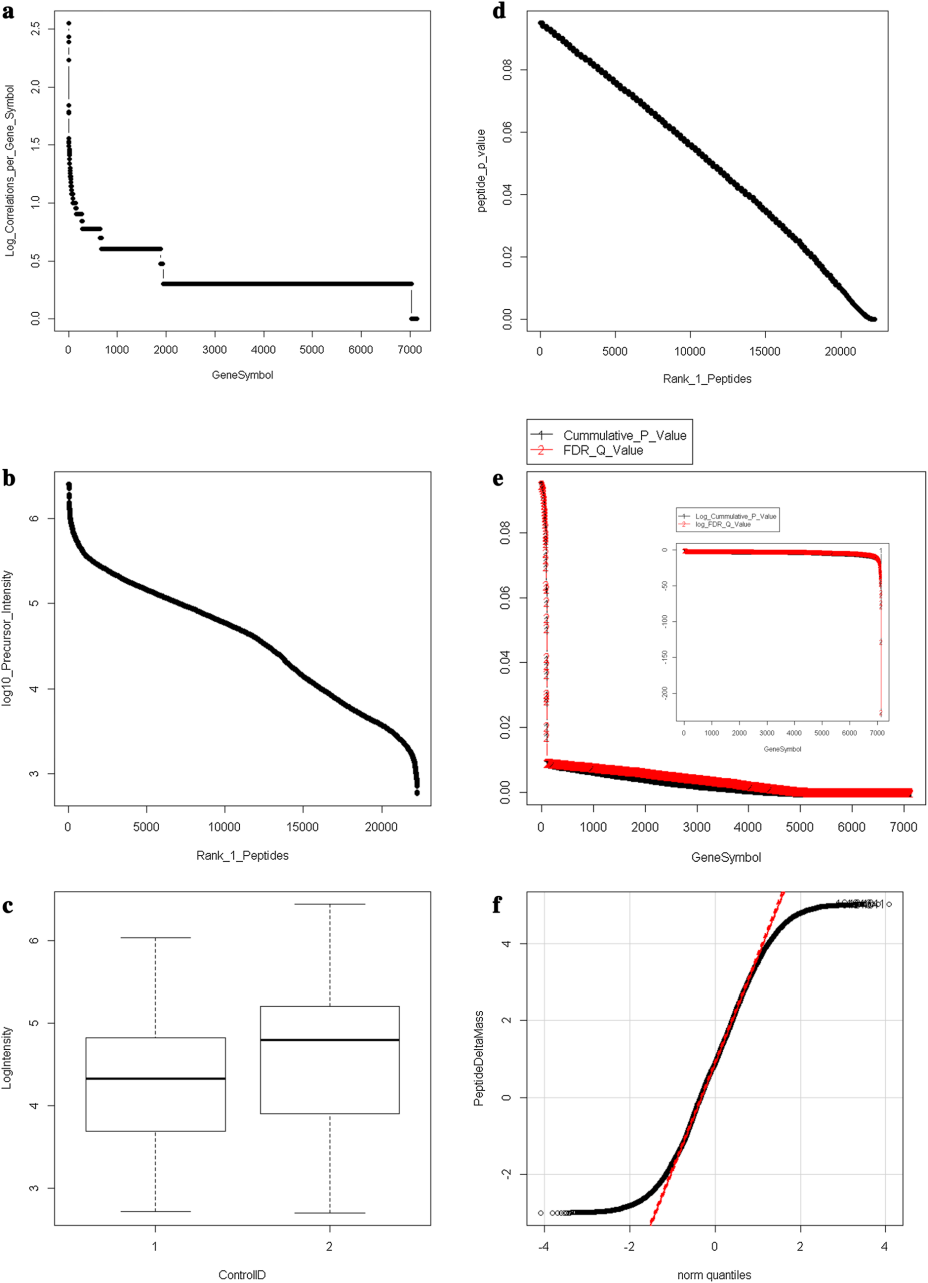
Statistical analysis of the 22,287 RANK1 correlations by X!TANDEM to tryptic peptides and phosphopeptides. The redundant peptide correlations from X!TANDEM were filtered to only RANK1 peptides from the protein accession with the highest number of correlations in each Gene Symbol category. **a** Peptide counts per gene symbol; **b** the intensity distribution of the correlated peptides; **c** the intensity of the preserved (1) versus room temperature (2) samples; **d** the average p-value of the peptides for each gene symbol; **e** the cumulative p-value for each gene symbol (inset, the log10 cumulative p-value for each gene symbol); **f** the normal quantile plot of peptide delta mass. The Filtered (Filter 2) data can be found in Additional file 1, Additional file 2, Additional file 3, Additional file 4

**Fig. 2:**
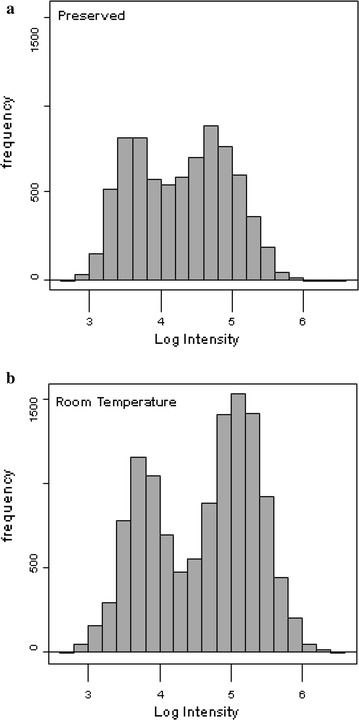
The Log_10_ Intensity distribution of 22,287 Density RANK1 correlations to tryptic peptide sequences with and without phosphorylation by X!TANDEM for preserved versus degraded samples. The RANK1 peptides from X!TANDEM were sorted into log10 intensity bins to compare preserved and degraded samples. **a** The frequency versus log intensity distribution of 82 preserved samples; **b** the frequency versus log intensity distribution of 88 room temperature samples. The Filtered (Filter 2) data can be found in Additional file 1, Additional file 2, Additional file 3, Additional file 4

The X!TANDEM algorithm was selected to examine the proteins in plasma on ice or at room temperature since it directly computes a p-value that the protein detected was merely random chance and has been compared to that of random spectra or noise for fully tryptic peptides from exogenous digestion [28–31]. The X!TANDEM algorithm fit MS/MS spectra to peptides in proteins but there may be multiple protein accessions that contain the same peptide sequence. One method to remove these redundant correlations to more than one protein accession number is to select the accession number with the highest number of correlations per gene symbol and then analyze the data at the peptide and protein level to yield a list of distinct RANK1 peptides. Starting with 583,927 unique MS/MS Spectra of > 1000 counts, the X!TANDEM algorithm correlated to some 181,962 peptide sequences and after selecting only the best scoring charge state (+ 2 or + 3) only 181,032 correlations remained. Once only the lowest p-value peptide sequence was accepted 94,669 redundant peptides potentially observed in more than 1 protein collapsed to 22,287 distinct RANK1 peptide observations in 7030 protein Gene Symbols with a 1% FDR and 3979 with a 0.02% FDR. The delta mass distribution was Gaussian from − 2 to + 2 Da (Fig. 1). There were many more high-intensity peptides observed in the room temperature versus the preserved EDTA plasma (Fig. 2). The list of Gene Symbols identified with the cumulative p-value and the FDR corrected q-value are provided in the Additional file 1, Additional file 2, Additional file 3, Additional file 4.

### X!TANDEM filtering

The complex key system in the SQL Server database was used to filter out all but the best p-value MS/MS to peptide fit at either 2^+^ or 3^+^ such that no mass spectra was assigned to more than one peptide sequence at one charge state. Filtering out re-use of the MS/MS spectra at different charge states or peptides sequences to accept only the best fit of the data to Gene Symbols with 5 or more independent correlations to precursors of greater than 1000 detector counts to fully tryptic peptides by X!TANDEM provided robust statistical reliability in agreement with previous results [28–32]. Accepting only protein accessions with 5 or more independent peptides resulted in 28,580 correlations. However, many protein types are represented by more than one related protein accession sequence that share the same gene symbol and so filtering only the single accession with the most correlations per gene symbol results in 7174 remaining MS/MS correlations to peptides in 733 distinct Gene Symbols (protein types). The STRING algorithm further reduced the number of gene symbols to about 660 (Table 1). Manual annotation to discount contaminants from source noise, random MS/MS spectra, dust controls, and closely related proteins from large gene families such as collagens results in at least 510 protein types. The set of ≥ 510 ten gene symbols with at least 5 best-fit peptides by X!TANDEM showed an FDR q-value of E−9 (i.e. FDR = 0.000000001) to E−227.

**Table 1:** The filtering of MS/MS to peptide correlations by the X!TANDEM algorithm. The LC–ESI–MS/MS results from X!TANDEM were parsed into an SQL Server database and a complex key or SHA1-HASH algorithm was used to create a non-redundant list of the best fit MS/MS to peptides in terms of charge state and amino acid sequence

Unique MS/MS Spectra > 1000 counts	583,927
Correlations from X!TANDEM No Filter	181,962
Correlations to BestCharge state + 2 or + 3	181,032
Correlation to Best Peptide P value	94,669
Correlations to Protein greater ≥ 5 counts	28,580
Correlations to Gene Symbol ≥ 5 counts	7174
Distinct Gene Symbol from R	733
STRING Gene Symbols in Network	660
X!TANDEM Filter 2	≥ 510

The R data analysis system was used to determine the non-redundant list of Gene Symbols with five or more counts

#### Ice cold samples protein interactions

**Fig. 3:**
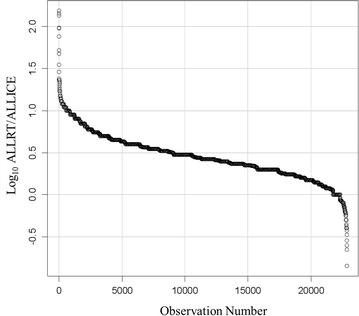
The filtered (best charge state 2^+^ or 3^+^ and peptide sequence) and sorted ratio peptide-to-protein counts (MS/MS correlations to peptides in a protein accession). The ratio of peptide-to-protein counts for each protein was computed from all room temperature samples (ALLRT) over all preserved samples (ALLICE) from a roughly equal number of LC–ESI–MS/MS runs for each group treatment. The proteins with the highest number of correlations in Room Temperature plasma (ALLRT) relative to samples preserved on Ice (ALLICE) was complement C4B. The Filtered (Filter 2) data can be found in Additional file 1, Additional file 2, Additional file 3, Additional file 4

Collecting EDTA plasma samples on ice and aliquoting the samples on ice resulted in low amounts of endogenous peptides as analyzed by LC–ESI–MS/MS. Proteins with at least 5 peptides from X!TANDEM showed that peptides from albumin were prominent in plasma collected on ice and never incubated at room temperature along with peptides from PLC, PTK2, PELP1, CACNA1A Zinc Finger Proteins, TRRAP (Transformation/transcription domain-associated protein) and others (Fig. 3 and Additional file 1, Additional file 2, Additional file 3, Additional file 4).

#### Room temperature incubation protein interactions

**Fig. 4:**
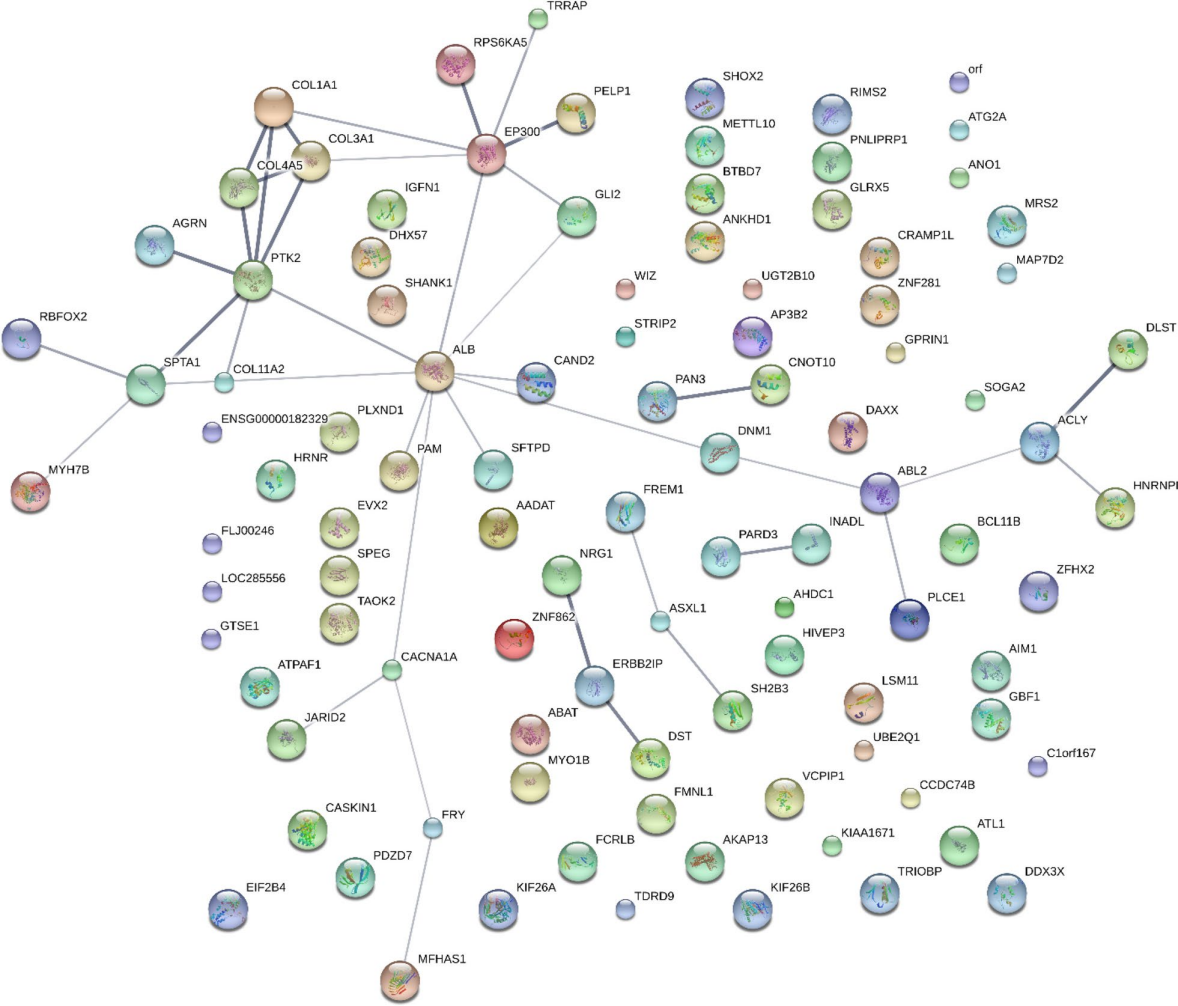
The filtered proteins of human plasma frozen or maintained on ice from at least 5 fully tryptic endogenously cleaved peptides as correlated by the X!TANDEM algorithm. The Network was produced using STRING confidence view. The Filtered (Filter 2) data can be found in Additional file 1, Additional file 2, Additional file 3, Additional file 4

Plasma has been shown to express a net weak tryptic activity towards exogenous protein substrates [45]. New peptides, and larger numbers of peptides, were observed from plasma proteins incubated at room temperature including many cellular proteins such as G-proteins, dyneins, myosin and actin associated filament proteins, kininogen, signalling enzymes such as small G proteins and other factors, such as nucleic acid binding proteins among many others (Fig. 4 and Additional file 1, Additional file 2, Additional file 3, Additional file 4).

### Analysis of X!TANDEM results across gene ontology terms

The set of 7174 proteins sequences with 5 independent peptides was collapsed by STRING to a set of 660 Gene Symbols that after manual annotation were examined in detail using the R generic statistical analysis system. To date about 74% of the 154,208 accession numbers in the Federated Human library have defined gene symbols. Where gene symbols exist they may be used to trigger the automated bioinformatics analysis by STRING. The STRING algorithm looks for previously established relations between the set of identified proteins [35, 36, 43]. The STRING algorithm tests to determine if the data set is merely a random assemblage of proteins or a network of proteins that show structural and functional relationships and so are known to exist and function together. The comparison of the distribution of proteins across category distributions provides a means to estimate the type I error rate of the data set [35, 36, 43]. The STRING analysis shows a previously established relationship with many of the processed proteins of human plasma including myosins, kininogen, leucine-rich repeat proteins, adenylate cyclase, EH Domain proteins Wiscott Aldrich Syndrome interacting protein WAS/WASL and dynactin that in turn show relationships to other preferentially degraded proteins. Multiple peptides from hundreds of cellular factors that are associated with human disease were observed in normal human plasma including phosphoinositide kinase, FYVE-finger-containing protein (PIKFYVE), serum/glucocorticoid regulated kinase (SGK3), monomeric GTPase activating proteins such as ARHGAP31 or RHOJ, Williams-Beuren Syndrome Chromosomal Region 18 Protein (DNAJC30), Zinc Finger proteins (ZNF), homeobox proteins and many other signalling and nucleic acid binding factors. The complete network derived from at least three peptides from X!TANDEM is too large to illustrate here and so is in the Additional file 1, Additional file 2, Additional file 3, Additional file 4.

#### Biological process

The endogenous peptides of normal human plasma were highly enriched in regulatory factors involved in morphogenesis, differentiation, neuron projection/development, cell organization, cellular organization or biogenesis and others to name a few (Table 2).

**Table 2:** The Distribution of the ≥ 510 gene symbols observed from the endogenous tryptic peptides of normal human plasma identified by X!TANDEM with respect to the human Gene Ontology (GO) Biological Processes as computed by STRING V10

Term	Number of genes	p-value	p-value_fdr	p-value_bonferroni
Multicellular organismal catabolic process	29	1.97E−27	1.71E−23	2.65E−23
Collagen catabolic process	28	2.55E−27	1.71E−23	3.43E−23
Collagen metabolic process	28	1.09E−25	4.90E−22	1.47E−21
Multicellular organismal metabolic process	29	2.76E−25	9.28E−22	3.71E−21
Multicellular organismal macromolecule metabolic	28	1.01E−24	2.72E−21	1.36E−20
Extracellular matrix organization	46	2.92E−22	6.34E−19	3.93E−18
Extracellular structure organization	46	3.30E−22	6.34E−19	4.43E−18
Extracellular matrix disassembly	29	1.28E−21	2.16E−18	1.73E−17
Neuron differentiation	56	1.36E−12	2.04E−09	1.83E−08
Neuron development	50	1.93E−12	2.60E−09	2.60E−08
Cellular component organization	151	8.25E−12	1.01E−08	1.11E−07
Axon guidance	34	2.42E−11	2.50E−08	3.25E−07
Cellular component organization or biogenesis	152	2.86E−11	2.75E−08	3.85E−07
Neuron projection morphogenesis	38	1.03E−10	9.22E−08	1.38E−06
Axon development	37	1.32E−10	1.11E−07	1.78E−06
Axonogenesis	36	1.50E−10	1.18E−07	2.02E−06
Cellular component disassembly	37	1.57E−10	1.18E−07	2.12E−06
Cell morphogenesis involved in neuron differentiation	37	1.98E−10	1.40E−07	2.66E−06
Generation of neurons	61	2.17E−10	1.46E−07	2.92E−06
Neurogenesis	63	2.85E−10	1.83E−07	3.84E−06
Neuron projection development	41	3.76E−10	2.30E−07	5.06E−06
Nervous system development	80	5.28E−10	3.09E−07	7.10E−06
Cell development	69	8.28E−10	4.64E−07	1.11E−05
Cell projection morphogenesis	41	1.81E−09	9.75E−07	2.44E−05
Cell part morphogenesis	41	4.63E−09	2.40E−06	6.23E−05
Collagen fibril organization	10	6.19E−09	3.08E−06	8.33E−05
Locomotion	55	7.21E−09	3.47E−06	9.70E−05
Chemotaxis	37	1.07E−08	4.80E−06	1.44E−04
Taxis	37	1.07E−08	4.80E−06	1.44E−04
Movement of cell or subcellular component	58	1.16E−08	5.02E−06	1.56E−04
System development	116	1.38E−08	5.81E−06	1.86E−04
Cell morphogenesis involved in differentiation	38	1.92E−08	7.81E−06	2.58E−04
Single-organism catabolic process	41	2.45E−08	9.70E−06	3.30E−04
Single-organism metabolic process	116	3.70E−08	1.42E−05	4.98E−04
Cell projection organization	45	9.88E−08	3.69E−05	1.33E−03

The headings are exactly as supplied by STRING. The Filtered (Filter 2) data can be found in Additional file 1, Additional file 2, Additional file 3, Additional file 4

#### Molecular function

The molecular functions of the proteins identified from the normal plasma peptides included a striking enrichment of proteins involved in ion and anion binding, adenyl ribonucleotide binding such as ATP/adenyl nucleotide binding, protein binding, carbohydrate binding, structural molecules and others (Table 3).

**Table 3:** The Distribution of the ≥ 510 gene symbols observed from the endogenous tryptic peptides of normal human plasma identified by X!TANDEM with respect to the human Gene Ontology (GO) Molecular Function as computed by STRING V10

Term	Number of genes	p-value	p-value_fdr	p-value_bonferroni
Extracellular matrix structural constituent	22	1.12E−19	4.39E−16	4.39E−16
Structural molecule activity	43	1.23E−13	2.43E−10	4.86E−10
Platelet-derived growth factor binding	7	3.19E−10	4.19E−07	1.26E−06
Carbohydrate derivative binding	77	7.96E−07	6.87E−04	3.13E−03
Adenyl ribonucleotide binding	59	1.04E−06	6.87E−04	4.08E−03
ATP binding	58	1.21E−06	6.87E−04	4.75E−03
Adenyl nucleotide binding	59	1.22E−06	6.87E−04	4.81E−03
Ion binding	167	1.78E−06	8.74E−04	6.99E−03
Extracellular matrix structural constituent conferring tensile strength	4	3.68E−06	1.61E−03	1.45E−02

The headings are exactly as supplied by STRING. The Filtered (Filter 2) data can be found in Additional file 1, Additional file 2, Additional file 3, Additional file 4

#### Cellular component

The normal human plasma was enriched in endogenous peptides from proteins of the extracellular matrix, cytoskeleton, basement membrane, microtubule, cytoplasm, organelles and others (Table 4). Proteins associated with membrane bound vesicles, secretion, exocytosis and exosomes were also observed.

**Table 4:** The Distribution of the ≥ 510 gene symbols observed from the endogenous tryptic peptides of normal human plasma identified by X!TANDEM with respect to the human Gene Ontology (GO) Cellular Component as computed by STRING V10

Term	Number of genes	p-value	p-value_fdr	p-value_bonferroni
Proteinaceous extracellular matrix	45	5.01E−24	7.94E−21	7.94E−21
Extracellular matrix	46	1.40E−22	1.11E−19	2.23E−19
Endoplasmic reticulum lumen	35	8.55E−22	4.51E−19	1.35E−18
Extracellular matrix component	28	3.25E−21	1.29E−18	5.15E−18
Collagen trimer	26	6.35E−21	2.01E−18	1.01E−17
Fibrillar collagen trimer	11	5.07E−17	1.34E−14	8.03E−14
Organelle lumen	153	2.79E−14	6.31E−12	4.42E−11
Intracellular organelle lumen	151	4.04E−14	8.00E−12	6.40E−11
Organelle part	220	5.07E−12	8.03E−10	8.03E−09
Basement membrane	16	3.64E−11	5.25E−09	5.77E−08
Intracellular organelle part	213	4.77E−11	6.30E−09	7.56E−08
Complex of collagen trimers	7	9.77E−11	1.19E−08	1.55E−07
Cytoplasm	255	1.75E−10	1.98E−08	2.78E−07
Organelle	277	1.33E−08	1.41E−06	2.11E−05
Endoplasmic reticulum part	51	3.26E−08	3.23E−06	5.17E−05
Intracellular organelle	259	4.64E−08	4.32E−06	7.35E−05
Intracellular	280	8.51E−08	7.49E−06	1.35E−04
Endoplasmic reticulum	62	1.59E−07	1.32E−05	2.52E−04
Cytoplasmic part	185	4.20E−07	3.33E−05	6.65E−04
Endomembrane system	108	4.90E−07	3.70E−05	7.76E−04
Intracellular part	276	5.95E−07	4.28E−05	9.42E−04
Membrane-bounded organelle	263	9.38E−07	6.46E−05	1.49E−03
Network-forming collagen trimer	4	1.25E−06	7.92E−05	1.98E−03
Collagen network	4	1.25E−06	7.92E−05	1.98E−03
Macromolecular complex	124	2.06E−06	1.25E−04	3.26E−03
Basement membrane collagen trimer	4	3.68E−06	2.16E−04	5.84E−03
Extracellular region part	112	5.58E−06	3.16E−04	8.85E−03
Protein complex	110	5.79E−06	3.16E−04	9.18E−03
Intracellular membrane-bounded organelle	238	6.50E−06	3.43E−04	1.03E−02
Collagen type V trimer	3	1.14E−05	5.81E−04	1.80E−02

The headings are exactly as supplied by STRING. The Filtered (Filter 2) data can be found in Additional file 1, Additional file 2, Additional file 3, Additional file 4

#### Tissues

The peptides detected by LC–ESI–MS/MS showed a strongly non-random distribution and were enriched in proteins that are expressed pleiotropically in all tissues and organs that should be expected to appear in plasma. Proteins observed in cells as well as from specific tissues including the ovary and internal female genital organs, the colorectum and large intestine, adrenal gland, spinal cord, gall bladder, heart, urinary bladder, and blood plasma among others were enriched (not shown).

#### Disease

Blood plasma was found to be enriched in peptides from proteins previously associated with disease, including monogenic, anatomical, autosomal, nervous system, musculoskeletal, recessive and sensory system disease (not shown).

#### Protein–protein interaction and pathway analysis

The proteins of normal human plasma as identified by the degradation products from endogenous tryptic activities were closely associated with KEGG pathways (Kyoto Encyclopedia of Genes and Genomes) such as extracellular matrix, focal adhesions as well as cell and calcium signalling (Table 5). A total of 1165 protein interactions were observed (expected 590) from 461 gene symbols that showed a probability of much less than p < 0.0001 [35].

**Table 5:** The Distribution of the ≥ 510 gene symbols observed from the endogenous tryptic peptides of normal human plasma with at least 5 peptides by X!TANDEM with respect to the human KEGG pathway as computed by STRING V10

Term	Number of genes	p-value	p-value_fdr	p-value_bonferroni
Protein digestion and absorption	26	7.78E−23	2.23E−20	2.23E−20
ECM-receptor interaction	19	3.79E−14	5.44E−12	1.09E−11
Focal adhesion	23	2.04E−10	1.95E−08	5.85E−08
Amoebiasis	16	1.47E−09	1.06E−07	4.22E−07
Platelet activation	16	2.51E−08	1.44E−06	7.22E−06
PI3K-Akt signaling pathway	25	2.02E−07	9.67E−06	5.80E−05

A total of 1165 protein interactions were observed (expected 590) from 461 gene symbols proteins that showed a probability of much less than p < 0.0001. The headings are exactly as supplied by STRING. The Filtered (Filter 2) data can be found in Additional file 1, Additional file 2, Additional file 3, Additional file 4

### Room temperature-specific proteins

**Fig. 5:**
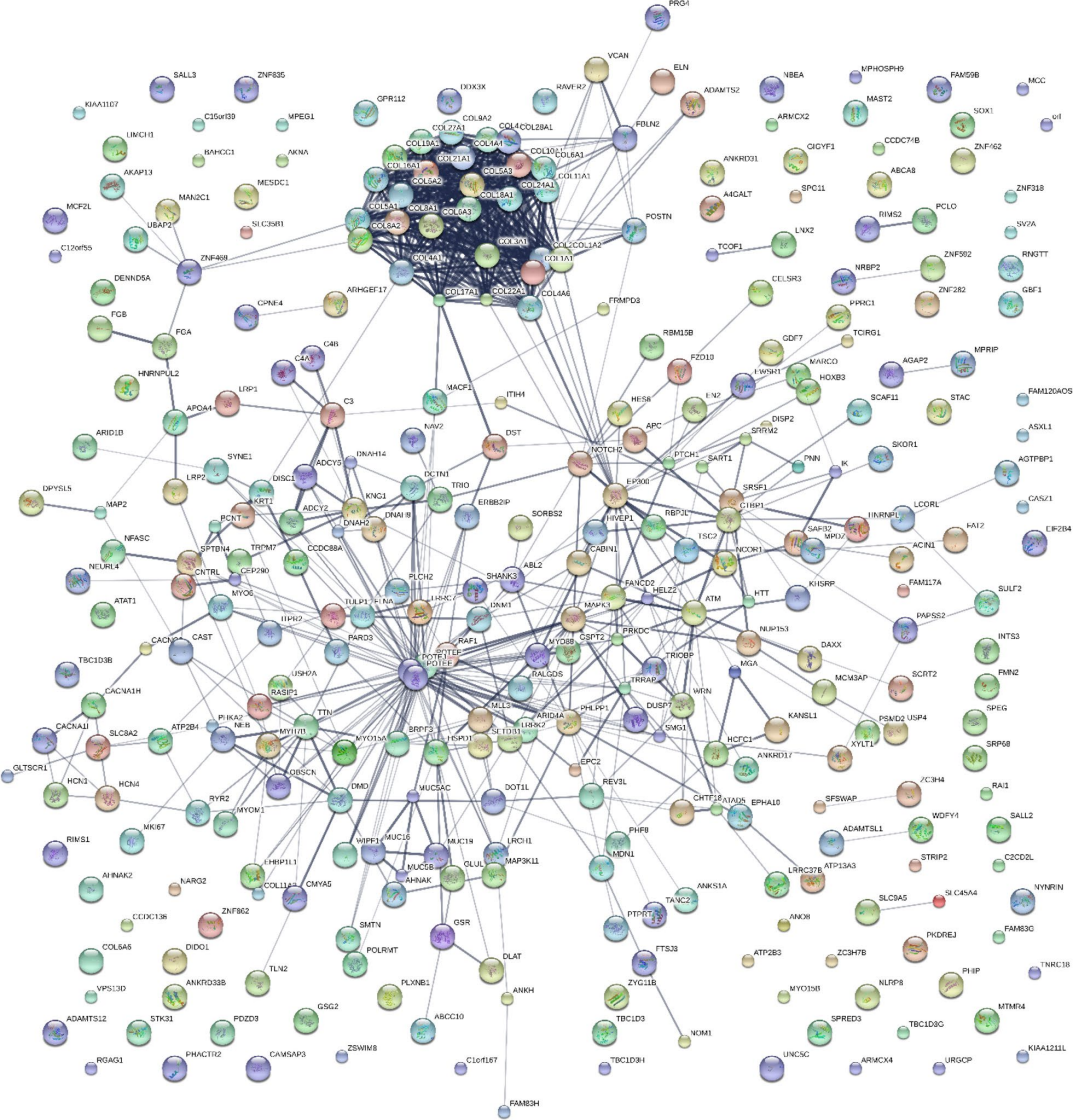
The protein interaction network of proteins from EDTA plasma that was incubated at room temperature detected from at least 5 fully tryptic endogenous peptides by the X!TANDEM algorithm. The Network was produced using STRING confidence view. The Filtered (Filter 2) data can be found in Additional file 1, Additional file 2, Additional file 3, Additional file 4

In contrast to samples maintained on ice over time, samples incubated at room temperature from 1 to 72 h showed some individual proteins such as C4B with 1 or 2 orders of magnitude more peptide correlations per protein (Fig. 5). Random mis-correlations from SEQUEST to giant proteins, noise and dust contamination are likely shared by all treatments to a roughly equal amount. Since false positive identifications should tend to be roughly equal in the room temperature versus ice cold samples, imposing a fivefold increase with temperature should tend to remove false positive identifications that should be common to both conditions. Thus, an alternative means to reduce the presence of random or noise mis-correlations to proteins was to compute the ratio of room temperature to ice cold samples that resulted in a list of 1171 gene symbols specific to room temperature including many cellular factors associated with membrane bound organelles, vesicle formation, exocytosis and vesicle targeting (Tables 6, 7, 8). The ratio of RT to ICE was computed for the proteins identified by SEQUEST where at least one peptide was also observed per gene symbol by X!TANDEM.

**Table 6:** Biological Process from the filtered (best charge state best amino acid sequence) X!TANDEM and SEQUEST results where the difference between room temperature versus ice cold samples was at least fivefold or greater for 718 gene symbols

Pathway ID	Pathway description	Count in gene set	False discovery rate
GO:0006996	Organelle organization	208	0.00138
GO:0016043	Cellular component organization	312	0.00138
GO:0071840	Cellular component organization or biogenesis	318	0.00138
GO:0009987	Cellular process	696	0.00194
GO:0008152	Metabolic process	558	0.0022
GO:0019538	Protein metabolic process	267	0.0022
GO:0044238	Primary metabolic process	505	0.0022
GO:0044237	Cellular metabolic process	496	0.00405
GO:0043170	Macromolecule metabolic process	441	0.00535
GO:0070887	Cellular response to chemical stimulus	166	0.00591
GO:0044260	Cellular macromolecule metabolic process	409	0.00645
GO:0071704	Organic substance metabolic process	510	0.00648
GO:0006464	Cellular protein modification process	185	0.0108
GO:0065007	Biological regulation	557	0.012
GO:0044763	Single-organism cellular process	573	0.015
GO:0048518	Positive regulation of biological process	314	0.0154
GO:0048585	Negative regulation of response to stimulus	103	0.0181
GO:0043412	Macromolecule modification	191	0.0223
GO:0009893	Positive regulation of metabolic process	229	0.0261
GO:0043933	Macromolecular complex subunit organization	150	0.0262
GO:0050789	Regulation of biological process	536	0.0262
GO:0044267	Cellular protein metabolic process	222	0.0341
GO:0008150	Biological_process	695	0.041
GO:0018193	Peptidyl-amino acid modification	72	0.0477

See the Additional file 1, Additional file 2, Additional file 3, Additional file 4 for the full list of proteins. The headings are exactly as supplied by STRING. The Filtered (Filter 2) data can be found in Additional file 1, Additional file 2, Additional file 3, Additional file 4

**Table 7:** Molecular Function from the filtered (best charge state best amino acid sequence) X!TANDEM and SEQUEST results where the difference between room temperature versus ice cold samples was at least fivefold or greater for 718 gene symbols

Pathway ID	Pathway description	Count in gene set	False discovery rate
GO:0005515	Protein binding	322	7.87E−05
GO:0003824	Catalytic activity	349	0.000801
GO:0005488	Binding	623	0.00118
GO:0043167	Ion binding	399	0.00118
GO:0005524	ATP binding	122	0.00244
GO:0003674	Molecular_function	731	0.00249
GO:0032559	Adenyl ribonucleotide binding	124	0.00249
GO:0000166	Nucleotide binding	172	0.00773
GO:0016887	ATPase activity	40	0.00773
GO:0044822	Poly(A) RNA binding	97	0.00868
GO:0008092	Cytoskeletal protein binding	50	0.0126
GO:0035639	Purine ribonucleoside triphosphate binding	138	0.0143
GO:0016740	Transferase activity	146	0.0186
GO:0032550	Purine ribonucleoside binding	137	0.0186
GO:0032553	Ribonucleotide binding	140	0.0186
GO:0032555	Purine ribonucleotide binding	139	0.0186
GO:0097159	Organic cyclic compound binding	353	0.0186
GO:1901363	Heterocyclic compound binding	348	0.0186
GO:0046914	Transition metal ion binding	110	0.0199
GO:0046872	Metal ion binding	271	0.0281
GO:0019899	Enzyme binding	102	0.03
GO:0043169	Cation binding	275	0.03
GO:0008270	Zinc ion binding	92	0.0498

See the Additional file 1, Additional file 2, Additional file 3, Additional file 4 for the full list of proteins. The headings are exactly as supplied by STRING. The Filtered (Filter 2) data can be found in Additional file 1, Additional file 2, Additional file 3, Additional file 4

**Table 8:** Cellular Component from the filtered (best charge state best amino acid sequence) X!TANDEM and SEQUEST results where the difference between room temperature versus ice cold samples was at least fivefold or greater for 718 gene symbols

Pathway ID	Pathway description	Count in gene set	False discovery rate
GO:0005737	Cytoplasm	682	5.45E−19
GO:0044424	Intracellular part	795	8.65E−16
GO:0005622	Intracellular	800	2.61E−13
GO:0044444	Cytoplasmic part	506	3.57E−12
GO:0043229	Intracellular organelle	707	6.75E−12
GO:0043226	Organelle	750	1.63E−11
GO:0043231	Intracellular membrane-bounded organelle	664	1.79E−11
GO:0044422	Organelle part	523	8.31E−11
GO:0043227	Membrane-bounded organelle	716	9.14E−11
GO:0044446	Intracellular organelle part	509	4.29E−10
GO:0005623	Cell	841	1.37E−09
GO:0044464	Cell part	839	1.37E−09
GO:0012505	Endomembrane system	250	5.36E−05
GO:0005575	Cellular_component	883	6.89E−05
GO:0031090	Organelle membrane	217	6.89E−05
GO:0043233	Organelle lumen	281	0.00025
GO:0005634	Nucleus	429	0.000297
GO:0005829	Cytosol	227	0.000297
GO:0031974	Membrane-enclosed lumen	282	0.000446
GO:0043232	Intracellular non-membrane-bounded organelle	243	0.000538
GO:0070013	Intracellular organelle lumen	272	0.0011
GO:0016020	Membrane	516	0.00164
GO:0031988	Membrane-bounded vesicle	237	0.00164
GO:0098588	Bounding membrane of organelle	162	0.00272
GO:0016023	Cytoplasmic membrane-bounded vesicle	84	0.00325
GO:0031982	Vesicle	240	0.00413
GO:0005794	Golgi apparatus	107	0.0054
GO:0044428	Nuclear part	244	0.00708
GO:0031410	Cytoplasmic vesicle	88	0.00869
GO:0000139	Golgi membrane	55	0.0155
GO:0072562	Blood microparticle	16	0.0173
GO:0044431	Golgi apparatus part	67	0.02
GO:0031981	Nuclear lumen	222	0.0223
GO:0005759	Mitochondrial matrix	36	0.0233
GO:0043190	ATP-binding cassette (ABC) transporter complex	4	0.0278
GO:0070062	Extracellular exosome	188	0.0278
GO:0005783	Endoplasmic reticulum	113	0.0283
GO:0031838	Haptoglobin-hemoglobin complex	3	0.0293
GO:0032991	Macromolecular complex	283	0.0312
GO:0005730	Nucleolus	68	0.0368
GO:0044433	Cytoplasmic vesicle part	46	0.0415
GO:0012506	Vesicle membrane	38	0.0465
GO:0005654	Nucleoplasm	188	0.0478

See the Additional file 1, Additional file 2, Additional file 3, Additional file 4 for the full list of proteins. The headings are exactly as supplied by STRING. The Filtered (Filter 2) data can be found in Additional file 1, Additional file 2, Additional file 3, Additional file 4

## Discussion

### Linear ion trap mass spectrometry

High resolution and low resolution mass spectrometers have been compared for the analysis of peptides from human blood fluids that showed good agreement but the ion trap is more sensitive and more robust [3–5, 7, 8]. High resolution mass spectrometers are required for the use of accurate mass strategy [46], for the use of isotopic [47, 48] or isobaric strategies [49] or carbohydrate analysis [50]. Simple ion traps are sufficient for the identification of tryptic peptides from Eukaryotes and so are appropriate for the sampling of peptides from human samples that have been fractioned by chromatography [51–53]. The low error rate of an ion trap mass spectrometer for identifying tryptic peptides does not result from a high resolution measurement of the precursor m/z value (± 3 m/z) but rather from fitting the MS/MS fragmentation spectra (± 0.5 Da) by algorithms such as SEQUEST [41] and X!TANDEM [40]. The low error rate of the sensitive ion trap for proteins with multiple peptides is well established and shows that confidence builds with the number of peptides identified such that proteins with three or more independent peptides show a low error rate [28–31, 54, 55]. The masses of most of the amino acid residues in MS/MS spectra can be effectively resolved by measurement to 0.5 Da by a simple ion trap but a high resolution mass spectrometer does not perform this task much better in practical terms. Moreover, proteins identified by low resolution mass spectrometers have been confirmed by Western blots, siRNA, drugs, immune cyto staining or GFP fusions [3, 37, 38]. Robust and sensitive ion traps [39, 56] may not always resolve the isotopic forms (R ≥ 1000–3000) leading to a concern that more than one peptide might be isolated resulting in mis-correlation of the MS/MS spectra. However, pre-separation of proteins or polypeptides by organic solvents, partition chromatography (including bio-specific or affinity separation), or differential centrifugation effectively enriches a sub-set of polypeptides that prevents co-elution of multiple tryptic peptides with similar m/z values [30, 31, 44]. Where polypeptides were efficiently pre-fractionated prior to analytical separation for LC–ESI–MS/MS the probability of tryptic peptides from more than one protein with similar m/z values eluting at the same time is dramatically reduced. The analysis of 300,000 synthetic peptides [57] agrees with the use of random and noise MS/MS control spectra that the fit of experimental MS/MS spectra to the predicted fragmentation patterns is statistically excellent and thus the reliable basis of peptide identification. The recent paper of Zolg using 300,000 synthetic peptides [57] confirms the observed MS/MS spectra closely matches the theoretical and thus MS/MS spectra matching with a simple ion trap is apparently sufficient to identify and quantify tryptic peptides that show low type I error by statistical analysis with respect to random expectation from null models of noise and random MS/MS spectra [28–31, 54, 55]. If there was a concern for false positive identification, the proteins p-values generated by the goodness of fit of the experimental MS/MS spectra might be corrected by the bona fide FDR method of Benjamini and Hochberg [42]. However, given the low p-values presented here, the corrected q-values from the FDR calculated by the method of Benjamini and Hochberg [42] were highly significant.

### Agreement between algorithms

The aim of this study was to determine the nature of the proteins and protein complexes that are susceptible to degradation by comparing endogenous tryptic peptides from normal human EDTA plasma on ice versus plasma incubated at room temperature. There are multiple sources of experimental and algorithm error that must be computed efficiently using SQL Server and a classical statistical analysis system such as R to interpret the results of the LC–ESI–MS/MS experiments with excellence. Plasma has a net mild tryptic activity [45] but also shows both C and N terminal exopeptidase activity [8, 58] that might confound the results of tryptic correlations. It is well established that C and N terminal exopeptidases are active in blood plasma and these enzymes cleave amino acids from both ends of tryptic peptides [8]. The SEQUEST algorithm has been shown to provide a type I error rate of about 1% where three independent peptides correlate to the same protein in tryptic digests [55]. In agreement with previous statistical analysis, the results of X!TANDEM were found to be highly reliable. Thus, to provide a more complete list of plasma peptides for the Additional file 1, Additional file 2, Additional file 3, Additional file 4 that also limits error, we have selected the proteins identified by SEQUEST with at least 5 peptides where the proteins were also detected by X!TANDEM at least once (see Additional file 5: Figure S1, Additional file 6: Figure S2).

### Multiple correlations from the same MS/MS spectra

A large source of error in LC–ESI–MS/MS is the re-use of the same MS/MS spectra that is correlated to multiple peptide sequences. The multiple peptide sequences correlated to the same MS/MS spectra may be eliminated by using a complex key also known as an SHA1-HASH for each MS/MS peptide-protein identification to retain only the highest scoring charge state and peptide sequence in SQL Server. Hence, the use of the automated functions of the SQL Server Database has the profound effect of avoiding the re-use of MS/MS spectra in proteomics to lower type I errors.

### Reporting results per Gene Symbol

There is significant redundancy in the LC–ESI–MS/MS of peptides since the same peptide sequence might be observed in multiple isoforms of the same protein and multiple predicted protein sequences. Collapsing the identical peptides observed in homologous proteins to one representative gene symbol is a convenient way to represent proteomic data that reduces the redundancy from proteins that share overlapping amino acid sequences. The analysis of the results stored in SQL Server can be computed on a per Gene Symbol basis using R to eliminate redundancy from similar protein sequences. Previously we collapsed these multiple proteins into protein types by manual annotation [7], or mapping short sequences into longer representative sequences using SQL or BLAST [7, 44]. The Gene symbols associated with the protein variants may be used to efficiently summarize the LC–ESI–MS/MS data using the SQL Server/R data storage and statistical analysis system.

### Random mis-correlation, noise and contamination

The SEQUEST algorithm may result in the false positive identification of large proteins [33] such as Titin (TTN), Nebulin (NEB), Spectrin protein (SYNE1) and others from random or noise spectra [28] at a low frequency. However, when the results of many LC–ESI–MS/MS experiments are analyzed together the number of correlations to these contaminants may become a concern. Another concern is the presence of keratins, mucins like MUC5AC, and POTE that may be detected in laboratory dust. A direct experimental and practical means to limit type I error from these sources is to compare experimental results to random spectra, noise from blank LC–ESI–MS/MS runs, and laboratory dust samples as negative controls. Alternatively making a ratio of treatments to look for proteins that differ between treatments also appears to be an effective means to limit the false positive identification of large proteins that tend to show a ratio of ~ 1.0 between sample sets. We conclude that caution must be exercised when analyzing proteins on these known contaminant lists using SEQUEST to ensure the frequency of detection is much higher than background.

### X!TANDEM

X!TANDEM is known to be a rigorous MS/MS correlation algorithm that relies on the fragment pattern from the precursor peptide to match endogenous tryptic peptides to amino acid sequences [40, 59]. In agreement with previous results, the measured type I error rate of fully tryptic peptides from X!TANDEM was low and so is one means to assign identity to low abundance endogenous tryptic peptides from plasma with confidence [28, 29]. Here, the use of a physical noise filter of E3 counts, together with the significant fit of at least three peptides [55] by X!TANDEM led to the apparent identification of many cellular proteins in plasma that are provided in the Additional file 1, Additional file 2, Additional file 3, Additional file 4. The X!TANDEM algorithm indicates that a pool of cellular proteins, with some major plasma proteins, are degraded in plasma with incubation at room temperature. The results indicate that the cellular proteins released into plasma are not as stable as the major plasma proteins. The cumulative p-values and FDR corrected q-values from X!TANDEM indicate that peptides from 510 types of functionally or structurally related proteins [7, 44] can be accurately identified by a one-step C18 chromatography preparation.

### STRING analysis

The STRING algorithm revealed a complex set of relationships at the level of homology, transcriptional regulation, protein–protein interactions, protein families and signal pathways. In agreement with the significant p-values assigned by the X!TANDEM correlation algorithms to MS/MS sequence matches, the STRING algorithm indicated that the results are not a random assemblage of proteins. It has been previously established that the distribution of proteins across categories may be used to estimate the probability that the data set is no different than random expectation that may serve as an estimate of type I error of identification [35, 36]. Structural, functional and protein-interactions were revealed in the relationships between many of the degraded proteins of human plasma and a variety of cellular factors were observed that may have great biological significance. Myosins, kininogen, leucine-rich repeat proteins, adenylate cyclase, EH Domain proteins Wiscott Aldrich Syndrome interacting protein WAS/WASL and dynactin and their interactors as well as cellular signaling proteins such as PYK2 including regulatory proteins such as receptors, GTPases such as RHO, SH2 and SH3 domain containing proteins, signalling enzymes such as phospholipases or MAPK may be released into the plasma as protein complexes. Cytoskeleton associated Ankyrin itself from red blood cells has been previously shown to encode an activity that results in the cleavage of complement [60]. The endogenous peptides of normal human serum, including kininogen (KNG1), and Ankyrin Domain Family proteins such as POTEJ have been previously detected by DEAE [7]. The significant distributions across networks, GO terms, the significant protein-interactions, and the expectation of the MS/MS spectra to the predicted peptide sequences using goodness of fit by X!TANDEM all agree the data obtained was highly significant [3, 33, 34]. A significantly large number of reciprocal protein–protein interactions were observed between the set of proteins identified that essentially precludes high rates of false positive identification, and clearly indicates that some circulating protein complexes are susceptible to degradation by endogenous tryptic peptide activities soon after incubation at room temperature. The simplest explanation is that functionally or structurally related proteins and/or protein complexes were preferentially cleaved by endopeptidases within the plasma.

### Enrichment of tryptic peptides at room temperature

One simply strategy to avoid potential false positive correlations is to take the ratio of the room temperature (where tryptic proteases may act) versus ice cold samples (where tryptic protease activity is significantly reduced) thus largely removing the results of random mis-correlations and noise shared by both. Accepting only tryptic peptides that show a fivefold increase in correlation after incubation at room temperature using the SEQUEST and X!TANDEM algorithms results in a list of cellular proteins that are cleaved in plasma at room temperature. Since the apparently spurious mis-correlations to titin (TTN) or other giant proteins from SEQUEST are shared by all treatments, one strategy to avoid noise and mis-correlation from the sensitive SEQUEST algorithm is to select tryptic peptides that are specific to room temperature incubation thereby largely eliminating the bias towards erroneous identifications to giant proteins. Quantifying the endogenous peptides may represent a direct approach to monitor levels of cellular protein in the plasma between experimental treatments or physiological states while avoiding the interfering peptides from albumin, apolipoproteins and other common plasma molecules [7]. Peptides from KNG1 were observed at room temperature and it is known to play a role in the regulation of the complement cascade [61]. Alternative splicing of the KNG1 transcript generates the cysteine protease inhibitor, high-molecular-weight kininogen (HMWK), that functions in the regulation of plasminogen that activate**s** the complement system and blood coagulation, and in turn is cleaved by the enzyme kallikrein to produce bradykinin that regulates inflammatory responses [62]. Kininogen and Ankyrin have been previously observed from tryptic peptides from serum [7] and in degraded plasma samples [27].

## Conclusion

The results here showed agreement that LC–ESI–MS/MS of high intensity blood peptides via an electrospray source with a linear ion trap may confidently identify peptides from blood plasma that are associated with extracellular matrix, cellular factors, specific tissues and organs as well as known diseases. The Type I error from correlating experimental MS/MS spectra to those predicted for human tryptic peptides was limited by X!TANDEM or by taking the ratio of room temperature versus preserved samples that was supported by the comparison to the null model of random peptides and the non-random distribution across GO terms. The agreement between independent algorithms, the p-value of the peptides generated in X!TANDEM and the FDR q-value of the proteins generated by SQL Server/R, the ratio between experimental treatments, and the statistically significant structural and functional relationships between the proteins identified were all consistent with the veracity of the set of peptides and proteins identified from endogenous tryptic peptides by LC–ESI–MS/MS. The simplest model that explains all of the data collected here and elsewhere is that the major proteins of plasma such as albumin and apolipoproteins are relatively stable in plasma but that complexes of cellular proteins in circulation are more susceptible to degradation. A pool of cellular proteins and/or protein complexes were apparently degraded by a tryptic like protease activity soon after incubation of plasma at room temperature. The ex vivo artefacts of tryptic peptides released by endogenous endopeptidases may be used to identify and quantify plasma proteins. More than 500 proteins including cellular regulatory factors such as phospholipases, GTPases, Zinc finger proteins, lipases, and others may be directly monitored in human plasma by a one step C18 solid phase extraction followed by micro electrospray LC–ESI–MS/MS. It will apparently be possible to analyze the low abundance blood proteins using the artefacts of endogenous tryptic peptides by simple extraction with C18 followed by micro electrospray LC–ESI–MS/MS using the precursor m/z and resulting fragment ions from ≥ 22,000 endogenous tryptic peptides fit by X!TANDEM to ≥ 500 gene symbols presented in the Additional file 1, Additional file 2, Additional file 3, Additional file 4. Understanding and documenting the set of peptides cleaved from proteins that appear over the baseline immediately after sample collection is a pre-condition for the rational exploration of blood peptides across disease or other physiological states. Collecting EDTA plasma directly on ice essentially prevents the degradation of blood proteins.

## Additional files

**Additional file 1.** Cummulative P values per gene symbol FDR.

**Additional file 2.** Peptide P-values XTANDEM.

**Additional file 3.** DHP Pass 17 Supplemental XTANDEM GT 0.

**Additional file 4.** XTANDEM Pass 17 Peptides Per Gene Symbol.

**Additional file 5: Figure S1.** The proteins of human plasma from samples on ice plus samples at room temperature from at least 5 endogenously cleaved peptides as correlated by the X!TANDEM algorithm. The network was produced using STRING confidence view. The Filtered (Filter 2) data can be found in Additional file 1, Additional file 2, Additional file 3, Additional file 4.

**Additional file 6: Figure S2.** The proteins of human plasma from samples on ice plus samples at room temperature from at least one fully tryptic peptides as correlated by the X!TANDEM algorithm. The network was produced using STRING confidence view. The probability that so many protein–protein interactions could be obtained by random chance was estimated by STRING to be p ≤ 0.0001. The Filtered (Filter 2) data can be found in Additional file 1, Additional file 2, Additional file 3, Additional file 4.

## Abbreviations

ACN: Acetonitrile
RSG: Random spectra generator
