# Supplementary figures and images for: The proteins cleaved by endogenous tryptic proteases in normal EDTA plasma by C18 collection of peptides for liquid chromatography micro electrospray ionization and tandem mass spectrometry
Source: Clin Proteomics. 2017 Dec 2;14:39. doi: 10.1186/s12014-017-9174-9 (PMC5712186; doi:10.1186/s12014-017-9174-9)

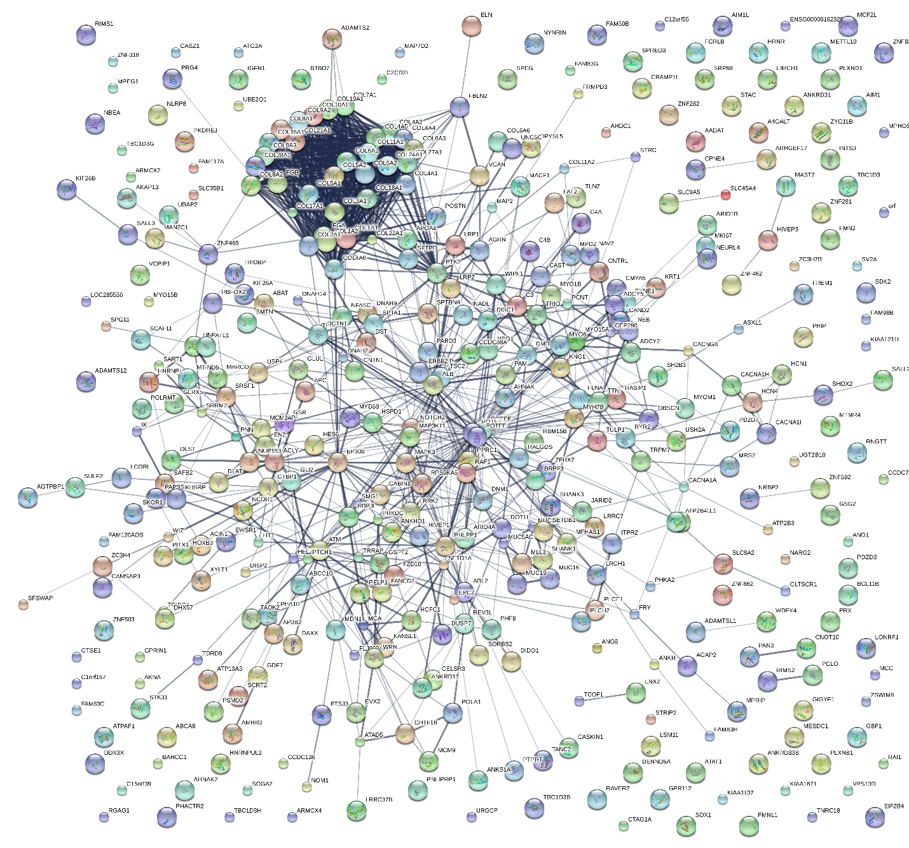

Supplement: Supplementary file 5 — Additional file 5: Figure S1. The proteins of human plasma from samples on ice plus samples at room temperature from at least 5 endogenously cleaved peptides as correlated by the X!TANDEM algorithm. The network was produced using STRING confidence view. The Filtered (Filter 2) data can be found in Additional file 1, Additional file 2, Additional file 3, Additional file 4. [file 12014_2017_9174_MOESM5_ESM.bmp]

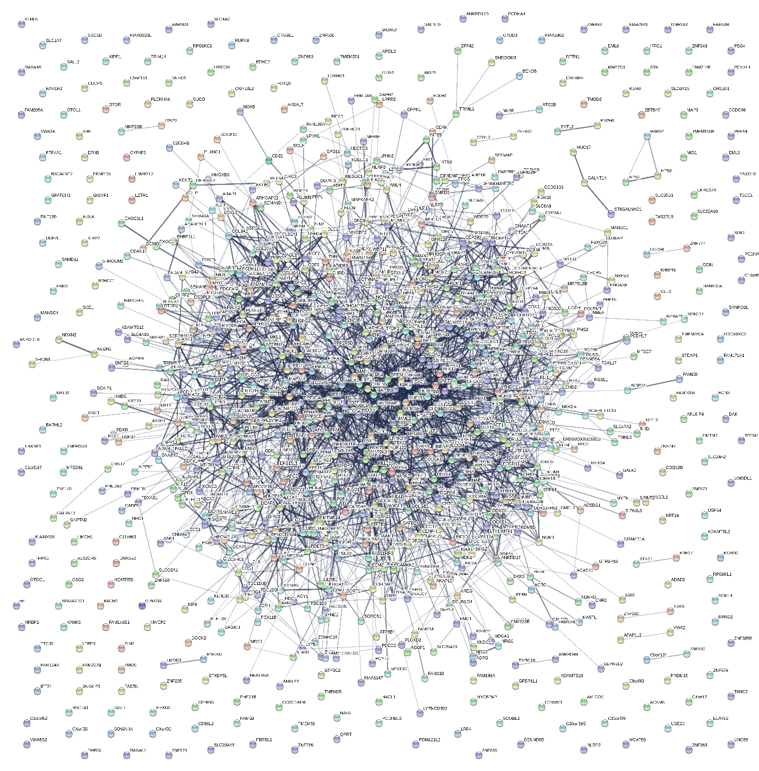

Supplement: Supplementary file 6 — Additional file 6: Figure S2. The proteins of human plasma from samples on ice plus samples at room temperature from at least one fully tryptic peptides as correlated by the X!TANDEM algorithm. The network was produced using STRING confidence view. The probability that so many protein–protein interactions could be obtained by random chance was estimated by STRING to be p ≤ 0.0001. The Filtered (Filter 2) data can be found in Additional file 1, Additional file 2, Additional file 3, Additional file 4. [file 12014_2017_9174_MOESM6_ESM.bmp]
